# 8-Bromo-3-phenyl-3a,4-dihydro-3*H*-chromeno[4,3-*c*]isoxazole-3a-carbo­nitrile

**DOI:** 10.1107/S1600536813002511

**Published:** 2013-01-31

**Authors:** G. Suresh, J. Srinivasan, M. Bakthadoss, S. Aravindhan

**Affiliations:** a Department of Physics, Presidency College (Autonomous), Chennai 600 005, India; b Department of Organic Chemistry, University of Madras, Chennai 600 025, India

## Abstract

In the title compound, C_17_H_11_BrN_2_O_2_, the five-membered isoxazole ring has an envelope conformation with the C atom bearing the phenyl ring as the flap. The pyran ring has a half-chair conformation. In the chromeno ring system, the dihedral angle between the mean plane of the pyran ring and the benzene ring is 4.68 (2)°. The dihedral angle between the mean planes of the chromeno ring system and the isoxazole ring is 13.79 (15)°. The latter forms a dihedral angle of 34.10 (17)° with the phenyl ring. In the crystal, mol­ecules are linked by C—H⋯N hydrogen bonds, forming an undulating two-dimensional network parallel to the *ab* plane.

## Related literature

For the biological importance of 4*H*-chromene derivatives, see: Cai (2007 ▶, 2008 ▶); Cai *et al.* (2006 ▶); Caine (1993 ▶); Gabor (1988 ▶); Brooks (1998 ▶); Valenti *et al.* (1993 ▶); Hyana & Saimoto (1987 ▶); Tang *et al.* (2007 ▶). For related structures, see: Gangadharan *et al.* (2011 ▶); Swaminathan *et al.* (2011 ▶). For puckering parameters, see: Cremer & Pople (1975 ▶).
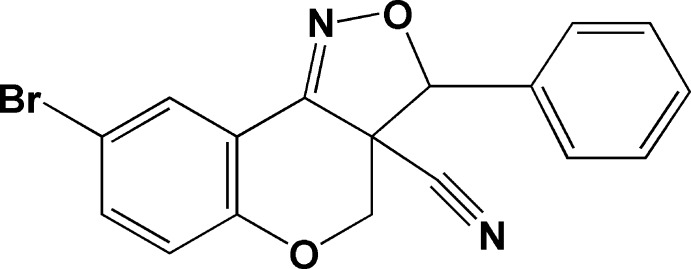


## Experimental

### 

#### Crystal data

C_17_H_11_BrN_2_O_2_
*M* *_r_* = 355.19
Monoclinic, 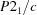

*a* = 15.1034 (8) Å
*b* = 6.0676 (3) Å
*c* = 16.0865 (10) Å
β = 99.953 (2)°
*V* = 1452.00 (14) Å^3^
*Z* = 4
Mo *K*α radiation
μ = 2.84 mm^−1^
*T* = 298 K
0.35 × 0.28 × 0.20 mm

#### Data collection

Bruker APEXII CCD area-detector diffractometer
Absorption correction: multi-scan (*SADABS*; Bruker, 2008 ▶) *T*_min_ = 0.437, *T*_max_ = 0.601
9947 measured reflections
3175 independent reflections
2214 reflections with *I* > 2σ(*I*)
*R*_int_ = 0.031

#### Refinement

*R*[*F* ^2^ > 2σ(*F* ^2^)] = 0.047
*wR*(*F* ^2^) = 0.102
*S* = 1.09
3175 reflections
199 parameters
H-atom parameters constrained
Δρ_max_ = 0.68 e Å^−3^
Δρ_min_ = −0.96 e Å^−3^

### 

Data collection: *APEX2* (Bruker, 2008 ▶); cell refinement: *SAINT* (Bruker, 2008 ▶); data reduction: *SAINT*; program(s) used to solve structure: *SHELXS97* (Sheldrick, 2008 ▶); program(s) used to refine structure: *SHELXL97* (Sheldrick, 2008 ▶); molecular graphics: *ORTEP-3 for Windows* (Farrugia, 2012 ▶) and *PLATON* (Spek, 2009 ▶); software used to prepare material for publication: *SHELXL97* and *PLATON*.

## Supplementary Material

Crystal structure: contains datablock(s) I, global. DOI: 10.1107/S1600536813002511/su2547sup1.cif

Structure factors: contains datablock(s) I. DOI: 10.1107/S1600536813002511/su2547Isup2.hkl

Supplementary material file. DOI: 10.1107/S1600536813002511/su2547Isup3.cml

Additional supplementary materials:  crystallographic information; 3D view; checkCIF report

## Figures and Tables

**Table 1:** Hydrogen-bond geometry (Å, °)

*D*—H⋯*A*	*D*—H	H⋯*A*	*D*⋯*A*	*D*—H⋯*A*
C2—H2⋯N2^i^	0.93	2.62	3.519 (5)	164
C15—H15⋯N2^ii^	0.93	2.61	3.334 (5)	135

Symmetry codes: (i) 
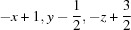
; (ii) 
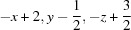
.

**Fig. 1.**
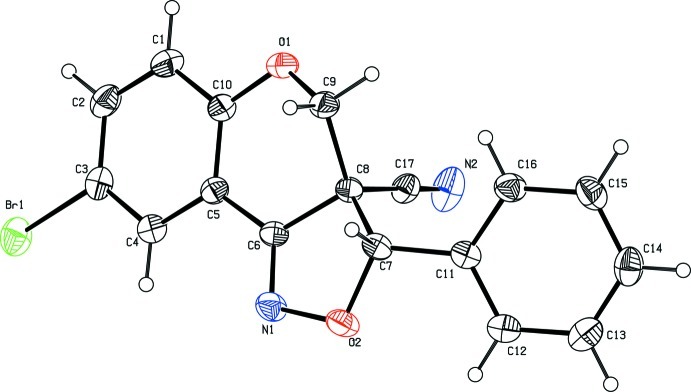
The molecular structure of the title molecule, with atom labelling. Displacement ellipsoids are drawn at the 30% probability level.

**Fig. 2.**
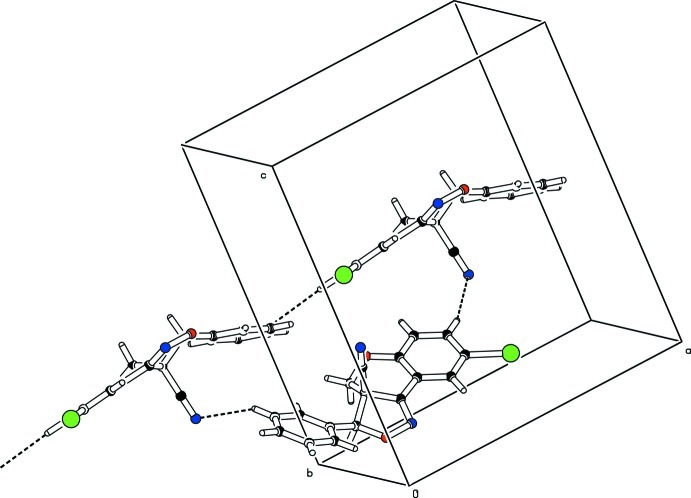
A view along the b axis of the crystal packing of the title compound. The molecules are linked by C—H···N hydrogen bonds (dashed lines; see Table 1 for details).

## supplementary crystallographic information

### Comment

4*H*-Chromenes are biologically important compounds used as synthetic ligands in the design of drugs and discovery processes. They exhibit numerous biological and pharmacological properties, such as anti-viral, anti-fungal, anti-inflammatory, anti- diabetic, cardionthonic, anti anaphylactic and anti-cancer activity (Cai, 2008, 2007; Cai *et al.*, 2006; Gabor, 1988; Brooks, 1998; Valenti *et al.*, 1993; Hyana & Saimoto, 1987; Tang *et al.*, 2007). Chromenopyrrole compounds are used in the treatment of impulsive disorders (Caine, 1993). Continuing our interest in such compounds we have synthesized the title compound and report herein on its crystal structure.

The molecular structure of the title molecule is illustrated in Fig. 1. The five-membered isoxazole ring (N1/O2/C7/C8/C6) adopts an envelope conformation with atom C7 as the flap: puckering parameters (Cremer & Pople, 1975) being q2 = 0.28795 (2) Å and Φ = 143.4 (2)°. In the chromeno ring system, the dihedral angle between the mean plane of the pyran ring and the benzene ring is 4.68 (2)°. The dihedral angle between the mean planes of the chromeno ring system (fusion of benzene and pyran rings) and the isoxazole ring is 13.79 (15)°. The isoxazole ring mean planes forms a dihedral angle of 34.10 (17)° with phenyl ring (C11—C16). The dihedral angle between the chromeno ring system mean plane and this phenyl ring is 25.42 (13)°. The atom Br1 deviates by 0.0379 (5) Å from the chromeno ring mean plane (O1,C1—C6/C8—C10). The geometric parameters of the title molecule agree well with those reported for closely related structures (Gangadharan *et al.*, 2011; Swaminathan *et al.*, 2011).

In the crystal, molecules are linked by intermolecular C—H···N hydrogen bonds forming an undulating two-dimensional network parallel to the ab plane (Fig. 2 and Table 1).

### Experimental

To a solution of (*E*)-2-((4-bromo-2-((*E*)-(hydroxyimino)methyl)phenoxy)methyl) -3-phenylacrylonitrile (2 mmol) in CCl_4_ at 273 - 283 K was added pinch wise NCS (4 mmol) over 3 h. After Et_3_N (4 mmol) was added to the reaction mixture which was stirred at room temperature for 2 h. After completion of the reaction, the mixture was evaporated under reduced pressure and the resulting crude mass was diluted with water (15 ml) and extracted with ethyl acetate (3 × 15 ml). The combining organic layer was washed with brine (2 × 10 ml) and dried over anhydrous Na_2_SO_4_. The organic layer was evaporated and purified by column chromatography (silica gel 60–120 mesh; 7% EtOAc in hexanes) to provide the desired pure title product as a colourless solid. Single crystals suitable for X-ray diffraction were obtained by slow evaporation of a solution of the title compound in ethyl acetate at room temperature.

### Refinement

All the H atoms were positioned geometrically and constrained to ride on their parent atom: C—H = 0.93, 0.97 and 0.98 A for aromatic, methine and methylene H atoms, respectively, with *U*_iso_(H) = 1.2U_eq_(C).

### Crystal data

**Table:**

C_17_H_11_BrN_2_O_2_	*F*(000) = 712
*M**_r_* = 355.19	*D*_x_ = 1.625 Mg m^−^^3^
Monoclinic, *P*2_1_/*c*	Mo *K*α radiation, λ = 0.71073 Å
Hall symbol: -P 2ybc	Cell parameters from 3175 reflections
*a* = 15.1034 (8) Å	θ = 2.6–28.6°
*b* = 6.0676 (3) Å	µ = 2.84 mm^−^^1^
*c* = 16.0865 (10) Å	*T* = 298 K
β = 99.953 (2)°	Block, colourless
*V* = 1452.00 (14) Å^3^	0.35 × 0.28 × 0.20 mm
*Z* = 4	

### Data collection

**Table:**

Bruker APEXII CCD area-detector diffractometer	3175 independent reflections
Radiation source: fine-focus sealed tube	2214 reflections with *I* > 2σ(*I*)
Graphite monochromator	*R*_int_ = 0.031
ω and φ scans	θ_max_ = 28.6°, θ_min_ = 2.6°
Absorption correction: multi-scan (*SADABS*; Bruker, 2008)	*h* = −20→17
*T*_min_ = 0.437, *T*_max_ = 0.601	*k* = −7→6
9947 measured reflections	*l* = −21→20

### Refinement

**Table:**

Refinement on *F*^2^	Primary atom site location: structure-invariant direct methods
Least-squares matrix: full	Secondary atom site location: difference Fourier map
*R*[*F*^2^ > 2σ(*F*^2^)] = 0.047	Hydrogen site location: inferred from neighbouring sites
*wR*(*F*^2^) = 0.102	H-atom parameters constrained
*S* = 1.09	*w* = 1/[σ^2^(*F*_o_^2^) + (0.0261*P*)^2^ + 2.1957*P*] where *P* = (*F*_o_^2^ + 2*F*_c_^2^)/3
3175 reflections	(Δ/σ)_max_ = 0.001
199 parameters	Δρ_max_ = 0.68 e Å^−^^3^
0 restraints	Δρ_min_ = −0.96 e Å^−^^3^

### Special details

**Table:**

Geometry. All e.s.d.'s (except the e.s.d. in the dihedral angle between two l.s. planes) are estimated using the full covariance matrix. The cell e.s.d.'s are taken into account individually in the estimation of e.s.d.'s in distances, angles and torsion angles; correlations between e.s.d.'s in cell parameters are only used when they are defined by crystal symmetry. An approximate (isotropic) treatment of cell e.s.d.'s is used for estimating e.s.d.'s involving l.s. planes.
Refinement. Refinement of *F*^2^ against ALL reflections. The weighted *R*-factor *wR* and goodness of fit *S* are based on *F*^2^, conventional *R*-factors *R* are based on *F*, with *F* set to zero for negative *F*^2^. The threshold expression of *F*^2^ > σ(*F*^2^) is used only for calculating *R*-factors(gt) *etc*. and is not relevant to the choice of reflections for refinement. *R*-factors based on *F*^2^ are statistically about twice as large as those based on *F*, and *R*- factors based on ALL data will be even larger.

### Fractional atomic coordinates and isotropic or equivalent isotropic displacement parameters (Å^2^)

**Table:**

	*x*	*y*	*z*	*U*_iso_*/*U*_eq_	
Br1	0.36162 (2)	0.32449 (7)	0.91825 (3)	0.05691 (17)	
C1	0.5050 (3)	−0.1845 (7)	0.8336 (2)	0.0511 (10)	
H1	0.4920	−0.3179	0.8057	0.061*	
C2	0.4363 (2)	−0.0557 (7)	0.8528 (2)	0.0497 (10)	
H2	0.3770	−0.1023	0.8383	0.060*	
C3	0.4558 (2)	0.1418 (6)	0.8935 (2)	0.0409 (8)	
C4	0.5432 (2)	0.2124 (6)	0.9171 (2)	0.0388 (8)	
H4	0.5555	0.3452	0.9457	0.047*	
C5	0.6132 (2)	0.0817 (6)	0.8976 (2)	0.0347 (7)	
C6	0.7069 (2)	0.1465 (5)	0.9180 (2)	0.0327 (7)	
C7	0.8603 (2)	0.0893 (6)	0.9367 (2)	0.0350 (8)	
H7	0.8709	−0.0167	0.9834	0.042*	
C8	0.7744 (2)	0.0220 (5)	0.87679 (19)	0.0328 (7)	
C9	0.7483 (2)	−0.2215 (6)	0.8748 (2)	0.0442 (9)	
H9A	0.7887	−0.3049	0.8460	0.053*	
H9B	0.7545	−0.2768	0.9321	0.053*	
C10	0.5935 (2)	−0.1162 (6)	0.8556 (2)	0.0397 (8)	
C11	0.9454 (2)	0.1107 (6)	0.90087 (19)	0.0335 (8)	
C12	0.9955 (2)	0.3024 (6)	0.9084 (2)	0.0463 (9)	
H12	0.9750	0.4258	0.9336	0.056*	
C13	1.0765 (3)	0.3117 (7)	0.8784 (2)	0.0540 (10)	
H13	1.1104	0.4404	0.8846	0.065*	
C14	1.1064 (2)	0.1313 (7)	0.8398 (2)	0.0527 (10)	
H14	1.1606	0.1374	0.8199	0.063*	
C15	1.0561 (2)	−0.0576 (7)	0.8308 (2)	0.0522 (10)	
H15	1.0756	−0.1793	0.8038	0.063*	
C16	0.9764 (2)	−0.0679 (6)	0.8617 (2)	0.0456 (9)	
H16	0.9431	−0.1976	0.8560	0.055*	
C17	0.7720 (2)	0.1102 (6)	0.7912 (2)	0.0380 (8)	
N1	0.74127 (18)	0.2977 (5)	0.96850 (19)	0.0449 (7)	
N2	0.7691 (2)	0.1831 (7)	0.7260 (2)	0.0633 (10)	
O1	0.65812 (17)	−0.2523 (4)	0.83272 (17)	0.0518 (7)	
O2	0.83596 (15)	0.2996 (4)	0.96973 (16)	0.0481 (6)	

### Atomic displacement parameters (Å^2^)

**Table:**

	*U*^11^	*U*^22^	*U*^33^	*U*^12^	*U*^13^	*U*^23^
Br1	0.0352 (2)	0.0664 (3)	0.0693 (3)	0.00649 (18)	0.00961 (17)	0.0061 (2)
C1	0.052 (2)	0.045 (2)	0.056 (2)	−0.0149 (19)	0.0085 (18)	−0.008 (2)
C2	0.0376 (19)	0.057 (3)	0.053 (2)	−0.0119 (18)	0.0041 (17)	0.001 (2)
C3	0.0349 (17)	0.047 (2)	0.0410 (18)	0.0027 (16)	0.0061 (14)	0.0104 (17)
C4	0.0389 (17)	0.038 (2)	0.0381 (17)	0.0003 (15)	0.0040 (14)	0.0013 (16)
C5	0.0369 (17)	0.032 (2)	0.0340 (17)	−0.0008 (14)	0.0043 (14)	0.0040 (15)
C6	0.0357 (16)	0.027 (2)	0.0335 (16)	0.0036 (14)	0.0016 (13)	0.0014 (15)
C7	0.0383 (17)	0.032 (2)	0.0326 (17)	0.0088 (14)	0.0006 (14)	−0.0005 (15)
C8	0.0345 (16)	0.030 (2)	0.0336 (16)	0.0013 (14)	0.0045 (13)	−0.0002 (14)
C9	0.0441 (19)	0.030 (2)	0.060 (2)	0.0007 (16)	0.0144 (17)	−0.0027 (18)
C10	0.0440 (19)	0.035 (2)	0.0419 (19)	−0.0032 (16)	0.0114 (15)	−0.0004 (16)
C11	0.0320 (16)	0.036 (2)	0.0285 (15)	0.0068 (14)	−0.0057 (13)	0.0014 (14)
C12	0.053 (2)	0.039 (2)	0.046 (2)	0.0008 (18)	0.0048 (17)	−0.0044 (18)
C13	0.052 (2)	0.055 (3)	0.053 (2)	−0.012 (2)	0.0028 (18)	0.006 (2)
C14	0.0397 (19)	0.075 (3)	0.044 (2)	0.000 (2)	0.0071 (16)	0.008 (2)
C15	0.046 (2)	0.058 (3)	0.054 (2)	0.010 (2)	0.0119 (18)	−0.010 (2)
C16	0.0390 (18)	0.041 (2)	0.055 (2)	0.0029 (16)	0.0043 (16)	−0.0070 (19)
C17	0.0292 (16)	0.046 (2)	0.0365 (19)	−0.0046 (15)	−0.0017 (14)	0.0008 (17)
N1	0.0341 (15)	0.045 (2)	0.0549 (18)	0.0062 (14)	0.0051 (13)	−0.0105 (16)
N2	0.0420 (17)	0.095 (3)	0.049 (2)	−0.0085 (18)	−0.0012 (15)	0.022 (2)
O1	0.0490 (15)	0.0387 (15)	0.0700 (18)	−0.0083 (12)	0.0170 (13)	−0.0172 (14)
O2	0.0321 (12)	0.0495 (16)	0.0604 (16)	0.0037 (11)	0.0012 (11)	−0.0236 (13)

### Geometric parameters (Å, º)

**Table:**

Br1—C3	1.899 (3)	C8—C9	1.528 (5)
C1—C2	1.376 (5)	C9—O1	1.425 (4)
C1—C10	1.385 (5)	C9—H9A	0.9700
C1—H1	0.9300	C9—H9B	0.9700
C2—C3	1.373 (5)	C10—O1	1.377 (4)
C2—H2	0.9300	C11—C16	1.375 (5)
C3—C4	1.377 (5)	C11—C12	1.382 (5)
C4—C5	1.400 (5)	C12—C13	1.390 (5)
C4—H4	0.9300	C12—H12	0.9300
C5—C10	1.384 (5)	C13—C14	1.373 (6)
C5—C6	1.451 (4)	C13—H13	0.9300
C6—N1	1.275 (4)	C14—C15	1.369 (6)
C6—C8	1.512 (4)	C14—H14	0.9300
C7—O2	1.454 (4)	C15—C16	1.381 (5)
C7—C11	1.502 (4)	C15—H15	0.9300
C7—C8	1.532 (4)	C16—H16	0.9300
C7—H7	0.9800	C17—N2	1.132 (4)
C8—C17	1.472 (5)	N1—O2	1.427 (3)
			
C2—C1—C10	120.3 (4)	O1—C9—H9A	109.4
C2—C1—H1	119.9	C8—C9—H9A	109.4
C10—C1—H1	119.9	O1—C9—H9B	109.4
C3—C2—C1	119.6 (3)	C8—C9—H9B	109.4
C3—C2—H2	120.2	H9A—C9—H9B	108.0
C1—C2—H2	120.2	O1—C10—C5	123.2 (3)
C2—C3—C4	121.3 (3)	O1—C10—C1	116.7 (3)
C2—C3—Br1	120.2 (3)	C5—C10—C1	120.0 (3)
C4—C3—Br1	118.5 (3)	C16—C11—C12	118.6 (3)
C3—C4—C5	119.1 (3)	C16—C11—C7	119.3 (3)
C3—C4—H4	120.4	C12—C11—C7	122.1 (3)
C5—C4—H4	120.4	C11—C12—C13	120.4 (4)
C10—C5—C4	119.6 (3)	C11—C12—H12	119.8
C10—C5—C6	117.7 (3)	C13—C12—H12	119.8
C4—C5—C6	122.7 (3)	C14—C13—C12	120.1 (4)
N1—C6—C5	127.9 (3)	C14—C13—H13	119.9
N1—C6—C8	114.0 (3)	C12—C13—H13	119.9
C5—C6—C8	118.1 (3)	C15—C14—C13	119.7 (3)
O2—C7—C11	110.6 (3)	C15—C14—H14	120.2
O2—C7—C8	102.9 (2)	C13—C14—H14	120.2
C11—C7—C8	118.0 (3)	C14—C15—C16	120.1 (4)
O2—C7—H7	108.3	C14—C15—H15	119.9
C11—C7—H7	108.3	C16—C15—H15	119.9
C8—C7—H7	108.3	C11—C16—C15	121.1 (4)
C17—C8—C6	108.6 (3)	C11—C16—H16	119.5
C17—C8—C9	111.6 (3)	C15—C16—H16	119.5
C6—C8—C9	107.5 (3)	N2—C17—C8	178.2 (4)
C17—C8—C7	111.9 (3)	C6—N1—O2	108.2 (3)
C6—C8—C7	98.8 (2)	C10—O1—C9	117.3 (3)
C9—C8—C7	117.3 (3)	N1—O2—C7	107.7 (2)
O1—C9—C8	111.0 (3)		
			
C10—C1—C2—C3	0.4 (6)	C4—C5—C10—O1	178.8 (3)
C1—C2—C3—C4	−1.3 (5)	C6—C5—C10—O1	−0.1 (5)
C1—C2—C3—Br1	178.6 (3)	C4—C5—C10—C1	−0.3 (5)
C2—C3—C4—C5	1.3 (5)	C6—C5—C10—C1	−179.2 (3)
Br1—C3—C4—C5	−178.6 (2)	C2—C1—C10—O1	−178.8 (3)
C3—C4—C5—C10	−0.5 (5)	C2—C1—C10—C5	0.4 (6)
C3—C4—C5—C6	178.3 (3)	O2—C7—C11—C16	−175.9 (3)
C10—C5—C6—N1	−166.3 (3)	C8—C7—C11—C16	−58.0 (4)
C4—C5—C6—N1	14.9 (5)	O2—C7—C11—C12	6.3 (4)
C10—C5—C6—C8	12.9 (4)	C8—C7—C11—C12	124.3 (3)
C4—C5—C6—C8	−166.0 (3)	C16—C11—C12—C13	−1.3 (5)
N1—C6—C8—C17	−99.7 (3)	C7—C11—C12—C13	176.4 (3)
C5—C6—C8—C17	81.0 (4)	C11—C12—C13—C14	1.1 (6)
N1—C6—C8—C9	139.5 (3)	C12—C13—C14—C15	0.1 (6)
C5—C6—C8—C9	−39.8 (4)	C13—C14—C15—C16	−1.1 (6)
N1—C6—C8—C7	17.1 (4)	C12—C11—C16—C15	0.3 (5)
C5—C6—C8—C7	−162.1 (3)	C7—C11—C16—C15	−177.5 (3)
O2—C7—C8—C17	88.2 (3)	C14—C15—C16—C11	0.9 (6)
C11—C7—C8—C17	−33.8 (4)	C5—C6—N1—O2	179.0 (3)
O2—C7—C8—C6	−26.0 (3)	C8—C6—N1—O2	−0.2 (4)
C11—C7—C8—C6	−148.0 (3)	C5—C10—O1—C9	18.8 (5)
O2—C7—C8—C9	−141.0 (3)	C1—C10—O1—C9	−162.1 (3)
C11—C7—C8—C9	97.0 (4)	C8—C9—O1—C10	−47.8 (4)
C17—C8—C9—O1	−62.8 (4)	C6—N1—O2—C7	−18.5 (4)
C6—C8—C9—O1	56.1 (4)	C11—C7—O2—N1	155.3 (3)
C7—C8—C9—O1	166.2 (3)	C8—C7—O2—N1	28.4 (3)

### Hydrogen-bond geometry (Å, º)

**Table:**

*D*—H···*A*	*D*—H	H···*A*	*D*···*A*	*D*—H···*A*
C2—H2···N2^i^	0.93	2.62	3.519 (5)	164
C15—H15···N2^ii^	0.93	2.61	3.334 (5)	135

Symmetry codes: (i) −*x*+1, *y*−1/2, −*z*+3/2; (ii) −*x*+2, *y*−1/2, −*z*+3/2.

## References

[bb1] Brooks, G. T. (1998). *Pestic. Sci.* **22**, 41–50.

[bb2] Bruker (2008). *APEX2*, *SAINT* and *SADABS* Bruker AXS Inc., Madison, Wisconsin, USA.

[bb3] Cai, S. X. (2007). *Recent Patents Anticancer Drug Discov* **2**, 79–101. 10.2174/157489207779561462 18221055

[bb4] Cai, S. X. (2008). *Bioorg. Med. Chem. Lett.* **18**, 603-607.

[bb5] Cai, S. X., Drewe, J. & Kasibhatla, S. (2006). *Curr. Med. Chem.* **13**, 2627–2644. 10.2174/092986706778201521 17017915

[bb6] Caine, B. (1993). *Science*, **260**, 1814–1816. 10.1126/science.8099761 8099761

[bb7] Cremer, D. & Pople, J. A. (1975). *J. Am. Chem. Soc.* **97**, 1354–1358.

[bb8] Farrugia, L. J. (2012). *J. Appl. Cryst.* **45**, 849–854.

[bb9] Gabor, M. (1988). *The Pharmacology of Benzopyrone Derivatives and Related Compounds*, pp. 91–126. Budapest: Akademiai Kiado.

[bb10] Gangadharan, R., SethuSankar, K., Murugan, G. & Bakthadoss, M. (2011). *Acta Cryst.* E**67**, o942. 10.1107/S1600536811009731 PMC3099989 21754210

[bb11] Hyana, T. & Saimoto, H. (1987). Jpn Patent JP 621 812 768.

[bb12] Sheldrick, G. M. (2008). *Acta Cryst.* A**64**, 112–122. 10.1107/S0108767307043930 18156677

[bb13] Spek, A. L. (2009). *Acta Cryst.* D**65**, 148–155. 10.1107/S090744490804362X PMC2631630 19171970

[bb14] Swaminathan, K., Sethusankar, K., Murugan, G. & Bakthadoss, M. (2011). *Acta Cryst.* E**67**, o905. 10.1107/S1600536811009378 PMC3099941 21754179

[bb15] Tang, Q.-G., Wu, W.-Y., He, W., Sun, H.-S. & Guo, C. (2007). *Acta Cryst.* E**63**, o1437–o1438.

[bb16] Valenti, P., Da Re, P., Rampa, A., Montanari, P., Carrara, M. & Cima, L. (1993). *Anticancer Drug. Des.* **8**, 349–360. 8251042

